# PPARs in Calorie Restricted and Genetically Long-Lived Mice

**DOI:** 10.1155/2007/28436

**Published:** 2006-11-28

**Authors:** Michal M. Masternak, Andrzej Bartke

**Affiliations:** ^1^Departments of Internal Medicine, Geriatrics Research, School of Medicine, Southern Illinois University, Springfield, IL 62794, USA; ^2^Departments of Physiology, Geriatrics Research, School of Medicine, Southern Illinois University, Springfield, IL 62794, USA

## Abstract

Peroxisome proliferator-activated receptors (PPARs) are members of the nuclear receptors superfamily. The three subtypes, PPAR*α*, PPAR*γ*, and PPAR*β*/*δ*, are expressed in multiple organs. These transcription factors regulate different physiological functions such as energy metabolism (including lipid and carbohydrate metabolism), insulin action, and immunity and inflammation, and apparently also act as important mediators of longevity and aging. Calorie restriction (CR) is the most effective intervention known to delay aging and increase lifespan. Calorie restriction affects the same physiological functions as PPARs. This review summarizes recent findings on the effects of CR and aging on the expression of PPAR*γ*, *α*, and *β*/*δ* in mice and discusses possible involvement of PPARs in mediating the effects of murine longevity genes. The levels of PPARs change with age and CR appears to prevent these alterations which make “PPARs-CR-AGING” dependence of considerable interest.

## THE PPAR FAMILY

Peroxisome proliferator-activated receptors (PPARs) are members of the nuclear receptor superfamily that are ligand-dependent transcription factors. The activation of PPARs requires forming heterodimers with retinoid X receptors (RXRs), which allow binding to their specific peroxisome proliferator response elements (PPREs) [1]. By binding to specific PPREs in enhancer sites of targeted genes, PPAR/RXR heterodimers regulate their expression. PPAR genes are known to be expressed in different organs, including reproductive organs, major insulin target organs (liver, white adipose tissue, skeletal muscle), cardiac tissue, and other. PPARs have a wide spectrum of actions which include adipocyte differentiation, lipid metabolism, insulin sensitization, tissue injury and wound repair, inflammation, and immunity.

There are three known subtypes in the PPAR superfamily, each encoded by separate genes: PPAR*α*, PPAR*β*/*δ* (also known as PPAR*β* or PPAR*δ*), and PPAR*γ*. The most explored gene of this superfamily and the most adipose-specific is PPAR*γ*. There are two recognized isoforms of PPAR*γ*: PPAR*γ*1 and PPAR*γ*2. These isoforms are generated by alternative splicing and alternate translation initiation [2–4]. Although PPAR*γ* is the most recently cloned gene from PPARs, it quickly drew attention as a target receptor for thiazolidinediones (TZDs), the drugs used as insulin sensitizers in type 2 diabetic patients (5–8).

As its name implies, PPAR*α* was the first gene cloned from this family. PPAR*α* is mainly expressed in the liver, skeletal muscle, heart, and kidney. In these organs, it regulates a wide variety of target genes involved in cellular lipid catabolism. PPAR*α* alters the expression of genes encoding enzymes involved in the fatty acid metabolic pathway, which activate the regulation of fatty acids *β* and *ω*-oxidation. These effects are mediated by the presence of PPREs that are under transcriptional control of PPAR*α* in the promoter regions of genes coding for the enzymes involved in this metabolic pathway [5]. The activation of PPAR*α* in the heart induces accumulation of myocardial lipids that leads to other features of diabetic cardiomyopathy [6]. PPAR*α*-deficient mice have increased levels of total and HDL cholesterol [7].

The function of the third PPAR nuclear receptor, PPAR*β*/*δ*, is still somewhat unclear. There are some indications that PPAR*β*/*δ* is involved in lipid metabolism [8], and studies have shown that it plays an important role in epidermal maturation and skin wound healing [9, 10].

## CALORIE RESTRICTION AND PPARs

Calorie restriction (CR) is of wide interest in the study of aging. There are numerous studies showing that CR can improve the health of individuals and help protect them from disease. CR is also recognized as the most effective intervention known to delay aging and increase lifespan [11]. The precise mechanisms of CR action on aging and longevity are still not well established, but CR is known to reduce body weight and the levels of plasma insulin, IGF-1, GH, glucose, and thyroid hormone.

Calorie restriction is also known to alter expression of large number of genes involved in lipid metabolism and insulin signaling. Expression of many of the same genes is regulated by PPARs acting as transcription factors. This suggests a possibility that PPARs mediate the effects of CR or that CR and PPAR/RXRs heterodimers activate the same signaling pathways.

## ACTION OF PPARs ON INSULIN SIGNALING

The agonists for PPAR*α* and PPAR*γ* are widely used in diabetes. The study of rats fed a high-fat diet (HFD) indicated that PPAR*α* and PPAR*γ* agonists, WY14643 and pioglitazone, respectively, decreased glucose and leptin levels in plasma. The plasma levels of insulin and triglyceride were also reduced in rats treated with PPARs agonists in comparison to control animals; however, pioglitazone caused significantly greater reduction in comparison to PPAR*α*-agonist-treated and control rats [12]. However, activation of PPAR*γ* caused significant increase of body weight, which is opposite to CR action. PPAR*α* agonist did not alter body weight and more importantly caused significant decrease of visceral fat weight in comparison to control and pioglitazone-treated rats [12]. This indicates that pioglitazone improves insulin sensitivity more effectively than WY14643. However, weight gain caused by PPAR*γ* agonist is detrimental to the well-being of diabetic rats or humans.

## DIET

The functions and characterization of PPARs suggest that these nuclear receptors are strongly connected with the diet. There is considerable evidence that various diets can affect PPARs action in different organs and that the responses to diets can be mediated by their effects on PPARs expression.

### High-fat diet

High-fat diet is known to induce insulin resistance and promote type 2 diabetes in laboratory animals. In rats and mice HFD causes obesity and increases plasma insulin, glucose, and leptin levels. In HFD-fed rodents, PPARs agonists improve insulin sensitivity, presumably via activation of this nuclear receptor.

#### PPAR*α* and high-fat diet

Studies of PPAR*α*-null mice indicated that the deficiency of this nuclear receptor can protect from insulin resistance induced by HFD [13]. In this study the authors showed that HFD increases body weight and plasma insulin level but only in normal animals, with no alteration in PPAR*α*-null mice. Moreover, insulin tolerance test (ITT), glucose tolerance test (GTT), and the calculated insulin resistance index indicated that HFD caused insulin resistance in normal animals, with no alteration of insulin signaling in PPAR*α* deficient mice [13]. However, studies of PPAR*α*-null mice subjected to fasting indicated that PPAR*α* deficiency can cause severe hypoglycemia [14, 15]. Moreover, most of PPAR*α* target genes were not altered in the liver and heart of fasted PPAR*α*-null mice in comparison to normal controls [15]. The authors also reported that in PPAR*α* deficient animals fasting caused hyperketonemia, hypothermia, and increase in plasma levels of free fatty acids, which reflects inhibition of fatty acid uptake and oxidation [14]. Concluding, PPAR*α* participates in glucose homeostasis which may be important to prevent hypoglycemia under fasting condition or during exercise. However, long-term metabolic stress such as HFD could become negative for health by developing insulin resistance.

#### PPAR*γ* and high-fat diet

Similarly to PPAR*α* deficiency, PPAR*γ* deficiency in adipose tissue (PPAR*γ*-adiposeKO) was reported to protect from obesity and insulin resistance caused by HFD [16]. Under HFD, this tissue-specific PPAR*γ* deficiency increased glucose tolerance in comparison to control animals on HFD. Moreover, the levels of insulin and leptin were significantly decreased in HFD-treated, PPAR*γ*-adiposeKO mice in comparison to normal animals subjected to the same diet. Interestingly, the deficiency of PPAR*γ* in adipose tissue resulted in increased PPAR*γ* mRNA levels in the liver when compared to normal controls [16]. As stated by the authors, this model suggests that improved insulin sensitivity under HFD in PPAR*γ*-adiposeKO mice can coexist with increased expression of PPAR*γ* in the liver.

### Calorie restriction diet

**Figure 1:**
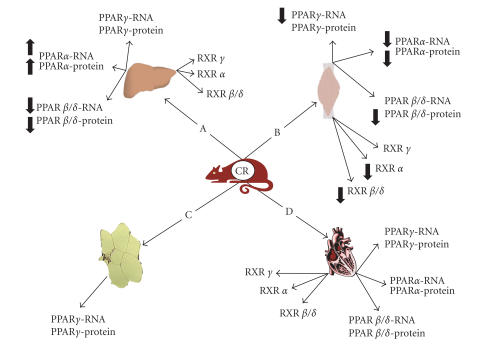
Effects of calorie restriction (CR) on the expression of PPARs family genes in mouse: (A) liver, (B) skeletal muscles, (C) white adipose tissue, and (D) heart. Arrows pointing up or down indicate statistically significant increases or decreases (*P* < .05). Lack of arrows means no alteration.

Calorie restriction is known to improve insulin sensitivity, lipid metabolism, health, and longevity. Calorie restriction is known to act on PPARs [17]; however, the effects are strikingly organ dependent. Depending on the organ, we observed a lack of changes, decrease or increase of PPARs expression in response to CR (Figure 1).

#### PPARs, CR, and the liver

Data reported by our laboratory indicated that 30% CR did not alter mRNA or protein levels of hepatic PPAR*γ* in mice. This finding suggested that improvement of insulin sensitivity in mice by CR is not mediated by PPAR*γ* in the liver [18, 19]. However, hepatic PPAR*α* mRNA and protein levels were significantly increased by CR in comparison to mice fed with unlimited (*ad libitum*; AL) access to food. This finding appears counterintuitive in view of the evidence that PPAR*α* deficiency prevents insulin resistance in mice subjected to HFD [13]. However, the suggested involvement of PPAR*α* in glucose homeostasis could imply that the increase of PPAR*α* in mice subjected to CR is a mechanism protecting these animals from hypoglycemia [14, 15]. Perhaps under conditions of HFD the decrease of PPAR*α* is adaptive, but when the animals are subjected to CR, PPAR*α* increases to facilitate maintenance of normal glucose levels during the periods when food is not available. Additionally, a recent study conducted by Corton et al indicated that 19% of hepatic genes involved in lipid metabolism, inflammation, and cell growth which were altered by CR were dependent on PPAR*α*. Interestingly, some of these genes were altered by CR only in normal mice but not in PPAR*α* deficient animals. Results obtained in animals treated with a PPAR*α* agonist indicated overlap of genes influenced by CR and by a compound activating PPAR*α* [20]. These important findings indicated that PPAR*α* plays an important role in mediating the action of CR [13, 20]. Corton et al also suggested that drugs activating the PPAR*α*-RXR-LXR axis can be potential CR mimetics [20].

The expression of the remaining member of the PPAR family, PPAR*β*/*δ*, in the liver was significantly decreased by CR at both mRNA and protein levels [19]. Thus, the hepatic expression of three genes from the PPAR family is differentially altered by CR. However, CR did not alter hepatic RXR*α*, RXR*γ*, and RXR*β*/*δ* mRNA (Figure 1A) [19].

#### PPARs, CR, and skeletal muscle

Similarly to the liver, the skeletal muscle is a major insulin target organ. In this tissue, the expression of PPARs and RXRs is altered differently by CR than in the liver [19, 21]. It was reported that 30% calorie restriction in mouse skeletal muscle decreased the level of PPAR*γ* mRNA and the PPAR*γ* protein level appeared to also be decreased [21]. We could speculate that the decrease of PPAR*γ* in the muscle as seen in the adipose specific knockout for PPAR*γ* is beneficial for insulin sensitivity [16]. However, muscle-specific knockout of PPAR*γ* caused whole-body insulin resistance [22]. Interestingly, treating these knockout mice with TZD improved insulin sensitivity [22], suggesting the effect was due to PPAR*γ* agonism in other tissues. This suggests that CR can increase insulin sensitivity through effects on PPAR*γ* expression in tissues other than the muscle, and speculating further we could suggest that under the conditions of CR, a decreased rather than elevated PPAR*γ* expression is beneficial.

PPAR*α* mRNA and proteins were decreased by CR in skeletal muscle, an effect opposite to that observed in the liver [19, 21]. It was speculated that a decrease of PPAR*α* in the muscle under CR slowed fatty acid oxidation, thus increasing the reliance on carbohydrates as the energy source. More importantly, consequences of reduced PPAR*α* expression could prevent the muscle from using all of the FFA immediately after food intake and thus maintain a balance between energy availability and energy usage during the fasting period. The protein level of PPAR*β*/*δ* was also decreased in the skeletal muscle of CR mice [21]. It is well known that CR promotes fat depletion and prevents obesity. Studies in PPAR*δ*-deficient mice on HFD revealed reduced energy uncoupling and obesity [21]. This would predict that reduced levels of PPAR*δ* in the muscles of CR mice may lead to increased lipid accumulation and promote obesity. However, reduced dietary fat intake in CR animals may alter these relationships. It was suggested that CR down-regulates the pathway of lipid metabolism and accommodates it to the circumstances of restricted food intake [21]. This may serve to prevent disruption of fatty acid homeostasis in CR animals. In addition to its effects on PPARs expression, CR reduced mRNA levels of RXR*α* and RXR*β*/*δ* [21]. Altered expression of these genes important to PPARs activation correlated with the changes in the expression of the corresponding members of the PPAR family (Figure 1B) [21].

#### PPARs, CR, and the white adipose tissue

PPAR*γ* is mainly expressed in white adipose tissue (WAT). As mentioned previously, the deficiency of PPAR*γ* in adipose tissue is protective against obesity and insulin resistance caused by HFD [16, 23]. However, CR increases insulin sensitivity in mice, without altering PPAR*γ* mRNA levels in WAT (Figure 1C) [24]. It can be speculated that under conditions of reduced calorie intake diminished PPAR*γ* would not be beneficial, or that limited fat storage does not allow increased PPAR*γ* activation in this tissue. At the time of this writing no data are available on the effects of CR on PPAR*α* and PPAR*β*/*δ* in WAT.

## PPARs IN GENETICALLY LONG-LIVED AND SHORT-LIVED MICE

### Growth hormone receptor/binding protein knockout (GHR-KO) mice

GHR-KO (Laron dwarf) mice have their GH receptor/binding protein gene disrupted and thus are deficient of GHR. Consequently these mice are GH resistant or insensitive and have greatly reduced plasma IGF-1 and insulin levels, and low glucose level [25, 26]. GHR-KO mice are also characterized by markedly extended lifespan in comparison to normal controls [27, 28].

**Figure 2:**
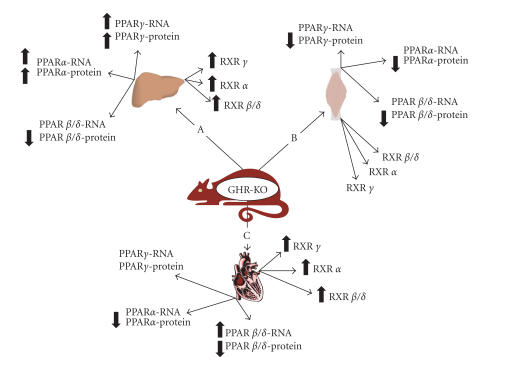
Effects of growth hormone receptor/binding protein knockout (GHR-KO) on the expression of PPARs family genes in mouse: (A) liver, (B) skeletal muscle, and (C) heart. Arrows pointing up or down indicate statistically significant increases or decreases (*P* < .05). Lack of arrow means no alteration.

In comparison to normal animals, GHR-KO mice also have significantly elevated PPAR*γ* mRNA and protein level in the liver (Figure 2A) [19]. We speculated that increased level of PPAR*γ* in the liver of those long-lived animals may be responsible for or contribute to their exceptionally high insulin sensitivity. This correlates with findings in PPAR*γ*-adiposeKO mice, which indicated that PPAR*γ* deficiency in WAT is compensated for by increased expression of this nuclear receptor in the liver to promote insulin sensitivity [16, 19]. The findings in the muscle also suggest that in GHR-KO mice PPAR*γ* in the liver contributes to high insulin sensitivity, because the level of PPAR*γ* mRNA in skeletal muscle of KO mice was not altered, while the PPAR*γ* protein level was decreased in comparison to normal controls (Figure 2B) [21].

The increased level of PPAR*α* measured in the liver of KO mice [19] could be suspect of exerting to negative effect on insulin action and obesity. However, the higher level of PPAR*α* suggests an increased usage of fatty acids, which could be beneficial for insulin sensitivity. Increased PPAR*α* levels could also be correlated with decreased total cholesterol level in GHR-KO animals [21]. It is interesting that the level of PPAR*α* in GHR-KO mice fed AL is maintained at the same level as in the normal animals subjected to CR. However, similarly to PPAR*γ*, expression of PPAR*α* was not altered in the muscle at the PPAR*α* mRNA level, while the PPAR*α* protein level was decreased in KO animals, again resembling the findings in normal mice under a CR regimen [21].

PPAR*β*/*δ* proteins were down-regulated in the liver and skeletal muscle of GHR-KO mice, which in both cases mimics the alterations of the expression of this gene caused by CR in the liver and muscle of normal mice [21].

These data indicate that CR alters PPAR*α* and PPAR*β*/*δ* proteins and/or mRNA levels in the liver and skeletal muscle to the levels maintained in GHR-KO animals. Since GHR-KO mice are long-lived and CR increases longevity, it can be suggested that PPAR*α* and PPAR*β*/*δ* play an important role in mediating the effects of both GHR-KO and/or CR on longevity. The RXR*γ*, RXR*α*, and RXR*β*/*δ* mRNA were increased in the liver and not changed in the muscle of GHR-KO mice which corresponds to alterations of PPAR*γ* and PPAR*α* in these two organs (Figure 2A, B) [19, 21].

#### Dwarf mice

Snell dwarf, Ames dwarf, and “Little” mice live markedly longer than their normal siblings. Snell dwarf mice carry a mutation in the Pit1 gene (Pit1^dw^) and Ames dwarf mice are homozygous for recessive loss-of-function mutation at the Prop1 locus (Prop1^df^). These dwarf mice are deficient with GH, prolactin, and tyrotropin. Little mice have severely reduced GH levels caused by the mutation of growth hormone-releasing hormone receptor (Ghrhr). Studies of Snell dwarf mice indicated increased hepatic PPAR*α* mRNA and protein levels in comparison to heterozygous controls [29]. The expression of PPAR*α* mRNA and protein in the liver of Ames dwarf mice was not altered in comparison to their normal controls [18]. However, gene array studies indicated that the genes regulated by PPAR*α* were either up-regulated in Snell dwarf, Ames dwarf, and Little mice or their expression increased in response to PPAR*α*-agonist treatment, which was interpreted as evidence that GH action is involved in the regulation of PPAR*α*-dependent gene products [29].

#### Phosphoenolpyruvate carboxykinase bovine-GH transgenic mice

PEPCK-bGH transgenic (TG) mice over-expressing the bGH gene fused to control sequences of the rat phosphoenolpyruvate carboxykinase (PEPCK) are characterized by markedly shortened lifespan in comparison to their normal siblings [30]. The findings in the liver did not indicate any alteration of PPAR*γ* mRNA in these TG mice. However, hepatic PPAR*α* mRNA was down-regulated in the liver of these short-living giant mice in comparison to their normal siblings [30]. This finding is very important in elucidating the potential role of PPAR*α* in the control of longevity. As mentioned above, PPAR*α* is increased in the liver of GHR-KO and CR mice. This contrasts with the decreased PPAR*α* level in the liver of short-lived b-GH transgenic mice. These findings are consistent with our suggestion that PPAR*α* can be an important mediator of genetic and dietary effects on longevity.

## AGING AND CALORIE RESTRICTION

As mentioned above, the study of PPAR*α*-null mice indicated that the deficiency of this nuclear receptor can protect from insulin resistance induced by HFD [13]. Furthermore, we speculated that PPAR*α* can be influential in the control of longevity, and we suggested that an elevated level of this nuclear receptor is beneficial and promotes longer life. Although PPAR*α* deficiency was useful in controlling glucose levels in HFD-fed mice, it was reported that these mice had age-dependent defects in heart, liver, and kidney, which correlated with decreased longevity in comparison to wild-type controls [31, 32]. PPAR*α* expression is also known to decrease with age in the liver, kidney, and heart. The study of GHR-KO and normal mice fed AL and subjected to CR indicated that PPAR*α* mRNA level in the heart is not affected by phenotype and CR (Figures 1D and 2C) [33]. CR increases the mRNA and protein level of PPAR*α* only in GHR-KO mice [33]. Interestingly, protein level of PPAR*α* was decreased in the heart of long-lived GHR-KO animals (Figure 2C) [33]. Moreover, earlier study [34] indicated that CR increased the cardiac level of PPAR*α* mRNA in mice, which would support antiaging action. Analysis of gene expression in mouse heart by Lee et al indicated that CR preserved fatty acid metabolism [35]. Additionally, the study of GHR-KO mice indicated that at the age of 3 months PPAR*α* was elevated in KO in comparison to the normal mice. When the animals reached 21 months of age this difference was no longer present [33]. The short-lived b-GH TG mice showed down regulation of this nuclear receptor in the heart at 9 months of age [33].

Sung et al reported that in rats the levels of PPAR*γ* and PPAR*α* in the kidney decreased with age when comparing 13- and 25-month-old animals [36]. Calorie restriction prevented these aging effects and maintained the levels of these nuclear receptors in 25-month-old rats at the same levels as in the 13-month-old animals [36]. To investigate the possible role of PPAR*α* and PPAR*γ* in inflammation, these authors also performed lipopolysaccharide (LPS) tests in young and old rats. Treatment with LPS decreased the level of these PPARs, to greater extent in old than in middle-age rats [36]. The authors concluded that down-regulation of PPARs in the rat's kidneys might be correlated with age-related oxidative stress, which could be reversed by antioxidative action of CR [36].

## SUMMARY

Different members of the PPARs family are expressed in many tissues. Various dietary regimens such as HFD and calorie restriction can affect expression of PPARs. However, the presence and direction of these changes depend on the organ being analyzed. Additionally, the effects of the diet on the animal depend on the actions of PPARs. For example, PPAR*α*-null or PPAR*γ*-adiposeKO mice are protected form insulin resistance and obesity caused by HFD. Studies in genetically long- or short-lived mice together with the studies involving CR suggest that PPARs play an important role in insulin action, lipid metabolism, immunity, and inflammation as well as regulation of aging and longevity.
